# A Case of Acute Respiratory Distress Syndrome Following Non-thoracic Trauma in a Patient With Idiopathic Pulmonary Fibrosis

**DOI:** 10.7759/cureus.63467

**Published:** 2024-06-29

**Authors:** Yukinori Hirooka, Soichiro Ota, Noriko Torizawa, Chihiro Maekawa, Youichi Yanagawa

**Affiliations:** 1 Acute Critical Care Medicine, Shizuoka Hospital, Juntendo University, Izunokuni, JPN

**Keywords:** hypoxia, fracture, acute respiratory distress syndrome, trauma, idiopathic pulmonary fibrosis

## Abstract

A 72-year-old man with idiopathic pulmonary fibrosis (IPF) was on home oxygen therapy at 1 L/min. He fell approximately 3 m onto a concrete surface while painting the roof of his home and was emergently transported to a local hospital due to pain in his lower back and right lower limb. His initial Krebs von den Lungen level decreased with medical treatments but has shown an increasing trend over the past three respiratory outpatient visits. His other medical conditions, including dyslipidemia, lumbar pain, and allergic rhinitis, were treated with several drugs prescribed by a nearby clinic. At the previous hospital, an increased oxygen demand of around 5 L via mask was noted, although other vital signs were stable. A plain whole-body computed tomography (CT) scan revealed pulmonary edema, a fracture of the right femoral neck, and a fracture of the third lumbar vertebral body. During transfer to our hospital for surgery, crossing the Amagi Pass at an elevation of approximately 830 m, the patient's respiratory condition rapidly deteriorated. Upon arrival, the cardiac wall movement was hyperdynamic, and PaO_2_ was 29 mmHg under supplemental oxygen at 15 L/min, necessitating oral endotracheal intubation and initiation of mechanical ventilation. A chest CT scan showed worsening diffuse ground-glass opacities in both lungs compared to the previous CT scan at the referring hospital. Despite positive pressure ventilation with the mechanical ventilator, the patient's condition did not improve, and he died in the emergency room.

Acute respiratory distress syndrome (ARDS) can occur following severe trauma but the onset of ARDS due to moderate trauma is extremely rare. Considering the possibility of an acute exacerbation of IPF prior to the injury, this report discusses the possibility of developing ARDS due to trauma-induced cytokines and lung damage from damage-associated molecular patterns, the possibility of inhaling dust while working on the roof, pneumonia caused by prescribed medication, viral infections, exposure to pollen and/or high altitude while passing through the mountain pass, and hypoxemia-inducing pulmonary edema.

## Introduction

Acute respiratory distress syndrome (ARDS) is characterized by sudden severe oxygen deficiency in the lungs, often with abnormal chest x-ray findings showing fluid in both lungs. This condition is not fully explained by heart failure or excess body fluid. ARDS can be triggered by various factors such as pneumonia, severe infection outside the lungs, aspiration, severe injury, pancreatitis, burns, inhaling smoke or toxic chemicals, drug overdose, coagulopathy, receiving multiple blood transfusions, or unstable circulation [1-3]. The initial insult leads to uncontrolled inflammation and activation of cytokine. Damage to the alveolar epithelial-endothelial barrier can occur directly from lung injuries, which primarily affect the lung tissue, or indirectly from injuries outside the lungs, which primarily affect the vascular endothelium due to widespread inflammation [1]. This damage causes fluid rich in proteins to leak into the lungs, disrupts the function of surfactant, and impairs the exchange of oxygen and carbon dioxide. ARDS can lead to changes in lung function, including reduced ability of the lungs to expand, increased areas of physiological dead space, and increased shunt. There is heterogeneity in outcomes among previous studies, with the 28-day mortality of ARDS remaining high, ranging from 10% to 67% [1]. 

Idiopathic pulmonary fibrosis (IPF) is a chronic fibrotic lung disease characterized by dry cough, fatigue, and progressive exertional dyspnea [4]. In this debilitating condition, lung parenchyma and architecture are destroyed, compliance is lost, and gas exchange is compromised. This leads inexorably to respiratory failure and death within 3-5 years of diagnosis. The etiopathogenesis of IPF is not fully understood, and treatment options are limited. Pathological features of IPF include extracellular matrix remodeling, fibroblast activation and proliferation, immune dysregulation, cell senescence, and the presence of aberrant basaloid cells [4].

We herein report a case of ARDS following moderate non-thoracic and non-head trauma in a patient with IPF, and discuss the mechanisms involved.

## Case presentation

A 72-year-old man with IPF under the care of our respiratory department, receiving home oxygen therapy at 1 L/min, fell from his home roof at a height of 2.5-3 m and sustained injuries. He was found by a neighbor who called for emergency assistance when he was unable to move. Five years ago, he was diagnosed with IPF after presenting with a treatment-resistant cough. At our hospital, his medication history included nintedanib 300 mg, L-carbocisteine 1500 mg, and lansoprazole 15 mg daily. Consequently, his initial Krebs von den Lungen (KL-6) level of 1409 U/mL (normal <500 U/mL) decreased to 504 U/mL, but had shown an increasing trend over the past three respiratory outpatient visits. His other medical conditions included dyslipidemia, lumbar pain, allergic rhinitis, and insomnia, for which he was prescribed atorvastatin, loxoprofen, levocetirizine, fluticasone propionate, carbetapentane, and dihydrocodeine/methylephedrine by a nearby clinic. He had no history of allergies, smoking, or drinking, and his activities of daily living were independent.

Upon contact with the paramedics, his SpO_2_ was 86% despite receiving oxygen at 3 L/min via nasal cannula, indicating poor oxygenation. He had mild tachycardia with a heart rate of 103 beats per minute, but his blood pressure was maintained. He was transported by ambulance to a nearby hospital, where a computed tomography (CT) scan revealed a fracture of the right femoral neck, a fracture of the third lumbar vertebral body, and pulmonary edema. He was then transferred to our hospital by ambulance for surgical intervention. During transport over the 834 m-high Amagi Pass, which is a mix of artificial forests including cedar and cypress trees, the patient experienced oxygen desaturation. Assisted ventilation with a bag valve mask (BVM) under high-concentration oxygen supplementation was initiated, but just before arrival at the hospital, SpO_2_ could not be measured. It took about 3 h from the time of injury to arrival at the hospital.

**Figure 1:**
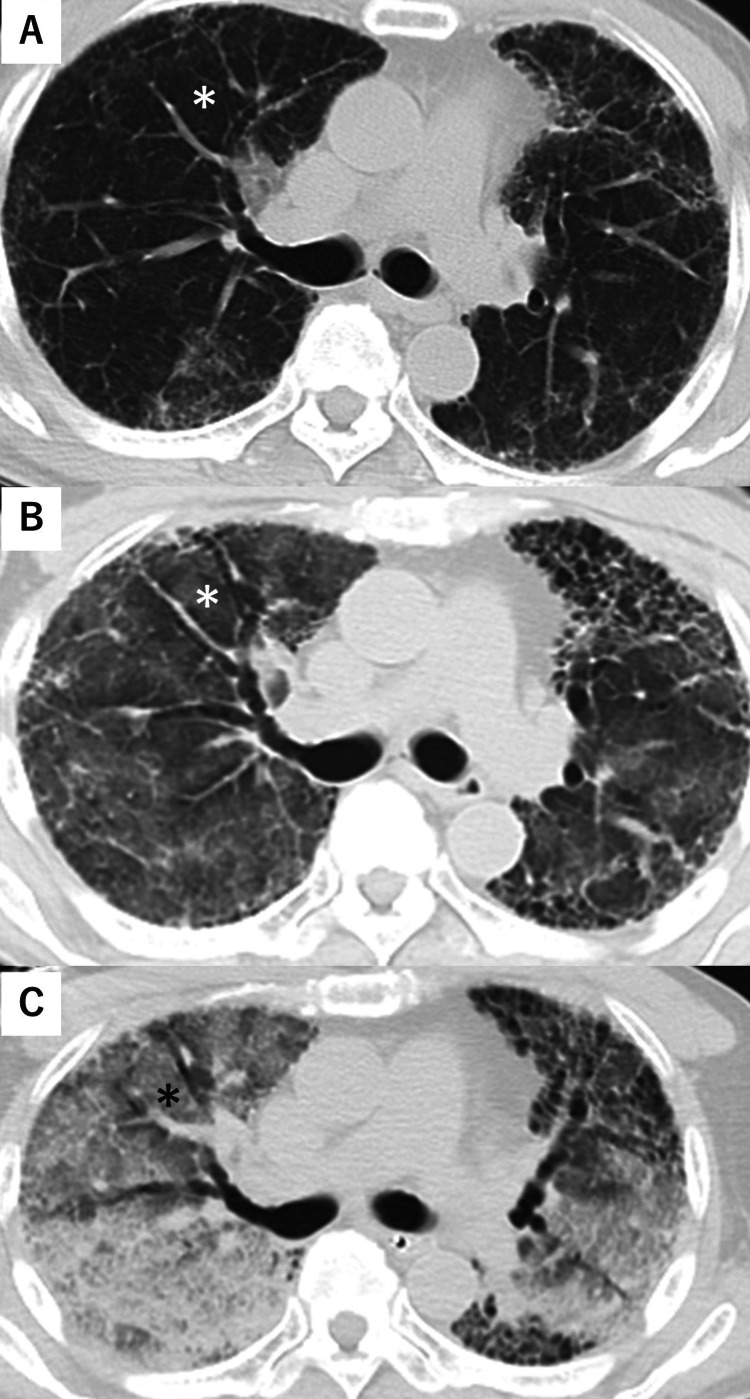
Chest computed tomography (CT) (upper [A]: 4 years before; middle [B]: referral hospital; lower [C]: on arrival) On the CT scan from four years ago, the ground-glass opacity is not clearly defined (A). At the referral hospital, diffuse ground-glass opacities appeared (B). After transfer to our hospital, the ground-glass opacity has worsened (C). Focusing on the right segment 3 (*), the difference is evident.

On arrival, the patient had a Glasgow Coma Scale score of E3V3M5, blood pressure of 195/126 mmHg, heart rate of 64 beats/min, SpO_2_ of 44% (under oxygen supplementation at 15 L/min with BVM ventilation), respiratory rate of 10 breaths/min, and a temperature of 36.8°C. On physical examination, pronounced coarse crackles were auscultated bilaterally. Arterial blood gas analysis (with oxygen at 15 L/min) showed a pH of 7.258, pCO_2_ of 46.9 mmHg, pO_2_ of 29.7 mmHg, HCO_3_^-^ of 20.2 mmol/L, and lactate of 7.6 mmol/L. Echocardiography revealed hyperdynamic movement and right ventricular dilatation. A 12-lead electrocardiogram showed atrial fibrillation. While preparing for emergency endotracheal intubation, the patient vomited frothy liquid. His pulse rate decreased, and there was a sudden decrease in cardiac wall motion, resulting in a near-cardiac arrest state. After stabilizing circulation with pressor agents, a plain and enhanced CT scan was performed. Ground-glass opacities in both lungs had worsened. The enhanced CT did not show obvious embolism in the pulmonary arteries (Figure 1).

The rapid tests for influenza and COVID-19 at the time of arrival were negative. The patient's laboratory results are shown in Table 1. 

**Table 1:** The patient's laboratory results

Item	Result	Unit
Total Protein	7.3	g/dL
Glucose	245	mg/dL
White Blood Cells	17.3 x 10^9^	/L
Albumin	3.1	g/dL
Blood Urea Nitrogen	7.9	mg/dL
Platelets	221.0 x 10^9^	/L
Total Bilirubin	1.3	IU/L
Creatinine	0.9	mg/dL
Alanine Aminotransferase	29	IU/L
Aspartate Aminotransferase	41	IU/L
Gamma-Glutamyltransferase	20	IU/L
Alkaline Phosphatase	96	IU/L
Lactate Dehydrogenase	591	IU/L
Creatine Phosphokinase	486	IU/L
Sodium	143	mEq/L
Potassium	3.8	mEq/L
Chloride	103	mg/dL
C-Reactive Protein	1.9	mg/dL
Brain Natriuretic Peptide	35.1	mg/dL
Prothrombin Time International Normalized Ratio	0.93	
Activated Partial Thromboplastin Time	24.7	s
Fibrinogen	296	mg/dL
Fibrinogen Degradation Products	88.5	μg/mL

Considering the pathophysiology of ARDS, management was initiated with a low tidal volume/high positive end-expiratory pressure ventilation strategy, but oxygenation did not improve. Additionally, CO_2_ gradually accumulated as seen in the arterial blood gas analysis, and the patient died in the emergency room. No autopsy was performed.

## Discussion

We discuss the mechanism of rapid deterioration in respiratory status during transport. First, regarding the possibility of cardiogenic pulmonary edema, the patient exhibited a hyperdynamic state on arrival according to cardiac ultrasound, no ST changes were observed on the 12-lead electrocardiogram, and brain natriuretic peptide (BNP) levels were almost within the normal range, leading to the conclusion that at least the respiratory function deterioration during transport was unlikely due to cardiogenic pulmonary edema [5,6]. Even if there is no decrease in ejection fraction, the possibility of diastolic heart failure (heart failure with preserved ejection fraction) remains. Unfortunately, in this case, the measurement of the E/Ea ratio (used for echocardiographic assessment of left ventricular filling pressure) was not performed [7]. However, it should be noted that in this case, BNP levels were barely elevated, blood pressure was usually not high, and the diffuse ground-glass opacity observed on the CT image was atypical for pulmonary edema caused by heart failure [8]. Therefore, the possibility of heart failure with preserved ejection fraction was considered low. Secondly, regarding respiratory dysfunction induced by trauma, there were no signs of chest contusions, and traumatic pulmonary edema due to direct blunt force trauma causing blood-gas barrier disruption was ruled out [9]. The possibility of fat embolism syndrome following trauma cannot be ruled out; however, it is extremely rare for respiratory function to deteriorate so rapidly with femoral neck or lumbar vertebral fractures. Additionally, the imaging indicates worsening pulmonary edema, which is not consistent with deterioration caused by fat embolism [10]. While respiratory status can deteriorate rapidly due to trauma-induced cytokines and lung damage from damage-associated molecular patterns, it is exceedingly rare for respiratory function to deteriorate so rapidly even with more severe trauma [11]. Thirdly, considering the possibility of acute interstitial pneumonia preceding the trauma, with the respiratory condition deteriorating rapidly during transport, it is not uncommon for endogenous diseases to precede trauma, especially in the elderly [12]. In the present case, there is a possibility of acute exacerbation of interstitial pneumonia complicating the underlying lung fibrosis. Previous reports indicate that 193 out of 1019 (18.9%) cases of lung fibrosis have experienced acute exacerbation. In this case, the patient has consistently shown elevated C-reactive protein (CRP) and lactate dehydrogenase (LDH) levels, and a gradual increase in KL-6 levels over the past three respiratory outpatient visits, suggesting an ongoing acute exacerbation. It is possible that the patient fell ill while working due to the acute exacerbation, despite no symptoms of collagen diseases such as rheumatoid arthritis or dermatomyositis being observed clinically. In addition, tests conducted three years ago showed negative rheumatoid factor, anti-cyclic citrullinated peptide antibody, and antinuclear antibody, ruling out ARDS due to acute interstitial pneumonia associated with collagen diseases [13]. While the possibility of developing ARDS from inhaling dust while working on the roof cannot be ruled out, and the possibility of pneumonia caused by medication prescribed at the hospital, COVID-19, and other viral infections besides influenza cannot be denied, the possibilities remain open [14,15]. Furthermore, due to the rapid deterioration in respiratory status during transport, there is a possibility that the patient was exposed to pollen while passing through the Mt. Amagi Pass, leading to a sudden worsening of respiratory status [16]. Additionally, while Mt. Amagi Pass is not a high-altitude pass, being less than 1000 m, which is not particularly problematic for healthy individuals, it may have had a negative impact on patients with respiratory distress [17]. Finally, it could be due to ARDS caused by hypoxemic pulmonary edema. It is clinically known that hypoxemia can increase pulmonary artery pressure, leading to the formation or exacerbation of pulmonary edema [18,19]. Although the patient's respiratory status deteriorated rapidly just before reaching the hospital, it is possible that hypoxemia-induced pulmonary edema exacerbated the condition. If the patient had received early tracheal intubation with a lung-protective strategy, such as low-tidal volume ventilation, permissive hypercapnia, and/or the use of neuromuscular blockers or steroids, he might have had a better chance of survival [19,20].

## Conclusions

We present a case of IPF who sustained a femoral neck fracture and a lumbar vertebral body fracture and experienced a rapid deterioration in respiratory function, leading to respiratory failure, during transfer to our hospital, resulting in death. Considering the possibility of an acute exacerbation of IPF prior to the injury, this report discusses the possibility of developing ARDS due to trauma-induced cytokines and lung damage from damage-associated molecular patterns, the possibility of inhaling dust while working on the roof, pneumonia caused by the prescribed medication, viral infections, exposure to pollen and/or high altitude while passing through the mountain pass, and hypoxemia-inducing pulmonary edema. When hypoxemia progresses rapidly, prompt intervention is essential to prevent further deterioration in respiratory status, highlighting the importance of vigilance in such cases.
